# Feeding a high-energy finishing diet upon arrival to high-risk feedlot calves: effects on health, performance, ruminal pH, rumination, serum metabolites, and carcass traits

**DOI:** 10.1093/jas/skac194

**Published:** 2022-05-27

**Authors:** David M Crawford, John T Richeson, Thomas L Perkins, Kendall L Samuelson

**Affiliations:** Department of Agricultural Sciences, West Texas A&M University, Canyon, TX 79016, USA; Department of Agricultural Sciences, West Texas A&M University, Canyon, TX 79016, USA; Department of Agricultural Sciences, West Texas A&M University, Canyon, TX 79016, USA; Department of Agricultural Sciences, West Texas A&M University, Canyon, TX 79016, USA

**Keywords:** body composition, cattle, energy, health, feedlot, receiving

## Abstract

This study evaluated the impacts of feeding a high-energy finishing diet during both the receiving and finishing period compared with a lower-energy receiving diet with adaptation to the finishing diet on health, performance, serum chemistry, ruminal pH, rumination, and carcass characteristics of high-risk feedlot cattle. Five truck-load blocks of steers (*n* = 101) and bulls (*n* = 299) were used in a generalized complete block design and randomly assigned to receive: 1) finishing diet for the entire feeding period (**FIN**) or 2) receiving diet for the first 56 d, followed by a transition to the finishing diet (**REC**). All cattle were fed ad libitum and consumed the same diet by day 74. A subset of cattle (*n* = 48) was randomly selected to quantify ruminal pH, temperature, and rumination time. Ultrasound images were collected on days 0, 74, and 146 to determine fat thickness over the 12th rib and rump, and carcass characteristics were determined after slaughter. Cattle fed FIN had less (*P* < 0.01) dry matter intake (**DMI**) from days 0 to 74, but DMI did not differ (*P* = 0.80) after day 74. From days 0 to final, DMI was 0.26 kg less for FIN compared with REC (*P* = 0.01). However, calculated metabolizable energy intake was not different from days 0 to 74 (*P* = 0.19), days 74 to final (*P* = 0.80), or overall (*P* = 0.78). Body weight (**BW**) on day 74 was greater (*P* < 0.01) and final BW tended to be greater (*P* = 0.10) for FIN compared with REC. Cattle consuming FIN had greater (*P* < 0.01) average daily gain and increased (*P* < 0.01) gain:feed from days 0 to 74. There were no differences (*P* ≥ 0.31) in health outcomes. On day 74, FIN had greater (*P* = 0.04) fat thickness over the rump and rib but did not differ (*P* ≥ 0.52) on day 146. Carcasses of FIN had greater (*P* = 0.04) hot carcass weight with no difference (*P* ≥ 0.11) in ribeye area, 12th rib fat thickness, yield grade, or quality grade. There was no difference (*P* = 0.18) in liver abscess rate. There was a diet × day interaction for blood urea nitrogen (*P* = 0.02) such that concentration decreased from days 0 to 28 in both treatments, but was less on day 28 for FIN. Ruminal pH was greater on days 2 and 61 and rumination time was less from days 0 to 28 for FIN (diet × day interaction; *P* < 0.01). Overall, these results suggest that providing a finishing diet fed ad libitum to high-risk calves upon arrival may be a viable alternative to a low-energy receiving diet.

## Teaser

High-risk cattle may not require a low-energy receiving diet containing high proportions of roughage upon arrival to the feedlot.

## Introduction

The term “high-risk” is used to classify cattle that experience limited pre-shipment management combined with chronic stress and likely exposure to novel pathogens before arriving at the feedlot, resulting in increased susceptibility to bovine respiratory disease (**BRD**). During the marketing process, these cattle are usually weaned and immediately transported to auction barns where they are comingled and have limited access to feed and water. Upon feedlot arrival, cattle experience additional stress such as handling, administration of implants, vaccines, and other animal health products, castration, and provision of unfamiliar feedstuffs that reduce their dry matter intake (**DMI**; Hutcheson and Cole, 1986) and alter the immune response (Taylor et al., 2010). However, high-risk cattle are typically offered hay and diets containing a greater proportion of roughage and lower energy than diets fed during the finishing period, despite the increased energy demand required to support inflammatory processes and low DMI. This phenomenon results in a concept previously described as the “receiving diet paradox” (Richeson et al., 2019).

Lofgreen et al. (1975) reported that providing greater dietary energy concentrations during the receiving period increased growth performance and morbidity of high-risk calves. Therefore, to stimulate DMI and mitigate the risk of BRD morbidity and ruminal acidosis, most high-risk cattle are provided a receiving diet with ≥30.0% roughage upon arrival (Samuelson et al., 2016). Ruminal acidosis is a metabolic disorder associated with the rapid fermentation of starch in feedlot diets that can decrease growth performance and DMI, and in severe cases, cause death (Owens et al., 1998). In addition to reducing dietary starch concentrations, roughage increases mastication and saliva production, which enhances buffering capacity that may reduce the prevalence of ruminal acidosis (Owens et al., 1998). Berry et al. (2004) observed numerically greater morbidity when cattle were fed diets with increasing starch and energy concentrations. However, cattle fed the high starch diet tended to have a lower percentage of *Pasteurella multocida* and *Histophilus somni* present in nasal swabs compared with cattle consuming diets with less starch and similar energy concentration. This suggests that perhaps cattle consuming high energy, high starch diets in the previous studies (Lofgreen et. al., 1975; Berry et al., 2004) may have been misdiagnosed for BRD because of the similarity of clinical signs of ruminal acidosis and BRD. Alternatively, increasing dietary energy intake may influence the immune response (Reuter et al., 2008).

Because of the increased availability of fibrous grain-milling products (**GMP**), starch concentrations have decreased in feedlot diets without greatly reducing energy. With the reduced starch concentrations afforded by GMP accompanied by the self-limited DMI of high-risk cattle, an opportunity may exist to provide a high-energy finishing diet containing GMP to high-risk, newly received cattle with less risk for ruminal acidosis and improved performance (Krehbiel et al., 1995). Tomczak et al. (2019) reported no difference in growth performance of newly received calves individually fed either a receiving or finishing diet containing GMP. However, health outcomes were not reported in the study conducted by Tomczak et al. (2019), and additional research is needed to evaluate how feeding a high-energy finishing diet after arrival affects the performance and health of feedlot cattle in a study with greater sample size. Our hypothesis was that providing a GMP-containing, high-energy finishing diet upon arrival to high-risk calves would result in greater growth performance and improved feed efficiency compared with a low-energy receiving diet with no effect on BRD morbidity. The study objective was to determine the effects of providing high-risk cattle ad libitum access to a GMP-containing high-energy finishing diet upon arrival compared with a low-energy receiving diet with a greater roughage concentration on growth performance, health, ruminal pH, rumination and activity time, serum chemistry, and carcass characteristics.

## Materials and Methods

All procedures involving live animals were approved by the West Texas A&M University Institutional Animal Care and Use Committee (2020.04.003).

### Cattle processing

A total of 400 high-risk beef calves (*n* = 299 bulls and 101 steers) with initial body weight (**BW**) of 252 ± 5.5 kg were purchased from auction markets in South Texas and shipped 735 km to the West Texas A&M University Research Feedlot on five different arrival dates (6 May 2020, 14 May 2020, 21 May 2020, 28 July 2020, and 19 August 2020). Immediately after arrival on day −1, cattle were individually weighed, affixed with a unique identification ear tag, ear notched to test for persistent infection with bovine viral diarrhea virus (CattleStats, Oklahoma City, OK), administered a clostridial vaccine with tetanus toxoid (Cavalry 9, Merck Animal Health, Kenilworth, NJ), *Mannheimia haemolytica* bacterin (Once PMH, Merck Animal Health), and a growth implant containing 36 mg zeranol (Ralgro, Merck Animal Health). One animal was removed from the study because it tested positive for persistent infection with bovine viral diarrhea virus. Additionally, cattle received metaphylactic treatment with tildipirosin (Zuprevo, Merck Animal Health), followed by a 7-d postmetaphylactic interval, and were treated for parasites with ivermectin, clorsulon (Ivermax, Aspen Veterinary Resources, Greely, CO), and albendazole (Valbazen, Zoetis, Kalamazoo, MI). All cattle were provided ad libitum access to long-stemmed hay and water overnight until the initiation of the experiment on day 0.

On day 0, cattle were individually weighed a second time to account for differences in gut fill and determine BW. Initial BW was considered the average of the days −1 and 0 BW. Additionally on day 0, bulls were castrated using a castration band (Callicrate Smart Bander, Callicrate Banders, St. Francis, KS) and orally administered 1 mg/kg BW of meloxicam (Unichem Pharmaceuticals, Hasbrouck Heights, NJ). The three median BW animals in each pen from the final two arrival dates (*n* = 48) were selected for blood collection and to receive a three-axis accelerometer ear tag (eSense flex tag, Allflex Livestock Intelligence, Madison, WI) and ruminal pH and temperature bolus (smaXtec pH Plus Bolus, SmaXtec, Graz, Austria). Accelerometer data measured total rumination and activity min within a 2 h period, while rumen boluses measured pH and temperature every 10 min. A pentavalent, modified live virus vaccine (Titanium 5, Elanco, Greenfield IN) was administered on day 28. Cattle were reimplanted on day 90 with a terminal growth implant containing 200 mg trenbolone acetate and 40 mg estradiol (Revalor XS, Merck Animal Health).

### Study design and treatments

This study was a generalized complete block design with five truckload blocks representing different arrival dates. Each block consisted of 4 pens per treatment with 10 animals per pen, resulting in a total of 20 pen replicates per treatment. Each pen was 27.4 × 6.10 m and offered approximately 48.3 cm of linear bunk space. The pen was considered the experimental unit. Cattle were stratified according to day −1 BW within each truckload and randomly assigned to pens such that the average BW and the number of bulls and steers were equalized between treatment pens. Experimental treatments were randomly assigned to pens on day 0 and consisted of: 1) lower energy, higher roughage receiving diet fed for the first 56 d of the study with a transition to a high-energy finishing diet over 18 d (**REC**; Table 1) or 2) a high-energy finishing diet containing GMP fed for the entire feeding period starting on day 0 (**FIN**). From day 74 to the end of the study (final), cattle were fed the same finishing diet. Diets were formulated to meet or exceed the nutrient requirements of growing and finishing beef cattle (NASEM, 2016) and 250 mg per animal ractopamine hydrochloride (Optaflexx, Elanco, Greenfield, IN) was provided in the diet for the final 34 d on feed (**DOF**).

**Table 1.** Composition and nutrient concentration of treatment diets

	Treatments	
Item	REC^1^	FIN^2^
Ingredient, % of DM		
Steam-flaked corn	24.2	62.0
Sorghum sudan hay	32.0	-
Corn stalks	-	8.0
Molasses blend^3^	5.0	2.5
Corn oil	-	3.0
Sweet Bran^4^	35.0	20.0
Supplement^5^	3.8	4.5
Nutrient analysis^6^, DM basis		
Dry matter, %	75.5	78.0
Crude protein, %	13.9	13.2
Acid detergent fiber, %	22.1	9.6
Crude fat, %	2.43	5.43
Total starch, %	25.9	52.5
ME^7^, Mcal/kg	2.76	3.20
NEm^7^, Mcal/kg	1.66	2.19
NEg^7^, Mcal/kg	1.05	1.51

REC, cattle fed a receiving diet for the first 56 d then transitioned to a finishing diet over 18 d.

FIN, cattle fed a finishing diet for the entire feeding period.

72 Brix Molasses Blend (Westway Feed Products LLC, Hereford, TX).

Sweet Bran (Cargill, Blair, NE).

Suplements were formulated to meet or exceed nutrient requirements for vitamins and minerals (NASEM, 2016) and mimic current feedlot practices (Samuelson et al., 2016). The REC supplement contained 15.8% urea, and provided 26.5 mg/kg monensin and 9.0 mg/kg tylosin and the FIN supplement contained 18.4% urea, and provided 38.0 mg/kg monensin and 9.0 mg/kg tylosin to the diet (Hi-Pro Feeds, Friona, TX).

Analyzed by Servi-Tech Laboratories, Amarillo, TX.

Metabolizable energy (ME), net energy for maintenance (NEm), and net energy for gain (NEg) were calculated from tabular values (NASEM, 2016).

### Feeding and health management

Feed bunks were visually assessed for a residual feed at 0630 and 2100 hours to determine the amount of feed to provide each day. Feed was provided once daily at approximately 0730 hours, and bunks were managed to allow less than 1 kg of feed remaining at the morning bunk reading. When greater than 1 kg of feed was visually estimated to remain in the bunk, the feed was removed, weighed, analyzed for DM, and used to calculate daily DMI. Bunk management between treatments was identical for the entirety of the study. On day 56, the dietary transition was initiated for the REC steers using a two-ration blending system where 10.0% of the daily feed allotment was replaced with 10.0% of the finishing diet every 2 d. The dietary transition period was used for cattle consuming REC to mimic current industry practices for newly received cattle (Brown et al., 2006; Samuelson et al., 2016). Both REC and FIN cattle were consuming the same diet beginning on day 74. Each diet was sampled twice weekly immediately after the feed was delivered. Diet samples were then divided into two portions. The first portion was analyzed for dry matter (**DM**; 100 °C for 24 h) on the day of collection in duplicate and used to calculate DMI, and the remaining sample was stored frozen (−20 °C) and composited twice monthly. Composited samples were analyzed by a commercial laboratory (Servi-Tech Laboratories, Amarillo, TX) for DM (using a two part drying process developed from NFTA 2.2.1.1. and NFTA 2.1.4), neutral detergent fiber (**NDF**; Ankom NDF Method 6.2017), acid detergent fiber (Ankom ADF Method 5.2017), crude protein (**CP**; AOAC 990.03), crude fat (**CF**; AOAC 920.39), and total starch (AOAC 996.11; AOAC, 2019).

Cattle were monitored daily by trained personnel for signs of BRD and assigned a clinical illness score (**CIS**) ranging from 0 to 4 as described by Pillen et al. (2016). In brief, a score of 0 indicated healthy animals, 1 indicated slightly ill animals, 2 indicated moderately ill animals that displayed multiple clinical signs of illness, 3 indicated severely ill animals that displayed multiple and severe signs of illness, and 4 indicated cattle that were moribund and near death. Cattle were removed from the pen and examined further if they had a CIS ≥ 2. Cattle were classified as morbid and treated with an antimicrobial if the rectal temperature was ≥40 °C and CIS ≥ 3 were treated regardless of rectal temperature. Antimicrobial treatment consisted of florfenicol (Nuflor, Merck Animal Health) for the first treatment, enrofloxacin (Baytril, Bayer Animal Health, Shawnee Mission, KS) for the second treatment, and ceftiofur crystalline free acid (Excede, Zoetis) as the third treatment. A posttreatment interval of 3-d was implemented after the first and second treatments. If cattle remained morbid after the third treatment and the prognosis of a full recovery was unlikely, cattle were removed from the study and considered chronically ill (*n* = 6).

Cattle were weighed before feeding at approximately 0600 hours on days −1, 0, 14, 28, 56, 74, and 174, and 2 consecutive days before harvest. Consecutive weights at the beginning and end of the study were averaged to mitigate differences in gut fill. Concomitant with BW measurements on days 0, 14, and 28, blood was collected via jugular venipuncture into evacuated tubes (BD Vacutainer SST, Becton Dickinson, Franklin Lakes, NJ) from the subset of animals determined on day 0. After blood was collected, it was centrifuged at 1,250 × *g* for 20 min, and the serum was decanted and stored at −20 °C for subsequent analysis. Serum chemistry concentrations of alanine aminotransferase (**ALT**), blood glucose, blood urea nitrogen (**BUN**), creatinine, sodium (Na^+^), potassium (K^+^), and chloride (Cl^−^), and total carbon dioxide (CO_2_) were determined using an automated analyzer (Abaxis VS2, Abaxis, Union City, CA). Twelfth rib fat thickness, rump fat, ribeye area, and marbling were assessed via ultrasound imaging on days 0, 28, 74, and 174 by an Ultrasound Guidelines Council certified technician using a New ALOKA SSD-500 console with a 3.5 MHz, 17.2 cm carcass probe (Corometrics Medical Systems, Wallingford, CT). Gain measurements on the ultrasound console were set at Mag = 1.5, overall gain = 90, near gain = −25, and far gain = 2.1. Before images were captured, the area to be imaged was shaved using hair clippers, cleaned using compressed air, and vegetable oil was applied as the couplant. A standoff pad, fitted to the transducer, was used when capturing the ribeye area images. Images were analyzed by the International Livestock Image Analysis laboratory (Harrison, AR).

Cattle were transported to a commercial abattoir (Tyson Fresh Meats, Amarillo, TX) when each block was estimated to have reached a shrunk BW of 635 kg. The average DOF of the 5 blocks was 269 d (minimum = 213 d and maximum = 302 d). After slaughter, hot carcass weight (**HCW**) and liver scores were recorded by a trained technician from the West Texas A&M Beef Carcass Research Center (Canyon, TX). After a 36-h chill, carcasses were assessed for fat thickness over the 12th rib, ribeye area, kidney-pelvic-and-heart fat, marbling score, and USDA quality grade. Yield grade and dressing percentage were subsequently calculated.

### Calculations

Metabolizable energy (**ME**) intake was calculated by multiplying the dietary ME concentration calculated from tabular values (NASEM, 2016) by DMI. During the transition period for REC, a weighted average that accounted for both the ME concentration of each diet and the proportion of each diet consumed was used to estimate dietary ME. All BW recorded in the study were adjusted using a 4.0% shrink. The dressing percentage was calculated by dividing HCW by the final shrunk BW (BW × 0.96). Carcass adjusted final BW (**AFBW**) was calculated by dividing HCW by a common dressing percentage of 64.65%. Carcass-adjusted ADG was determined using: carcass-adjusted ADG = (carcass-AFBW − initial shrunk BW)/DOF. Carcass-adjusted gain:feed (**G:F**) was determined by dividing the carcass-adjusted ADG by DMI. Calculated yield grade was determined using the USDA (1997) equation.

Calculated empty body fat (**EBF**) was determined using the equation: EBF = 17.76207 + (4.6812 × fat thickness) + (0.01945 × HCW) + (0.81855 × quality grade) − (0.06754 × ribeye area) as described by Guiroy et al. (2001). Similarly, empty BW (**EBW**) was also calculated from Guiroy et al. (2001), where EBW = (1.316 × HCW) + 32.29. Adjusted final BW was determined using the equation AFBW = [EBW + (28 − EBF) × 14.26]/0.891 as described by Guiroy et al. (2001). Performance-calculated net energy (**NE**) for maintenance (**NEm**) was calculated using the quadratic equation described by Zinn and Shen (1998), where NEm = −b ± √(b^2^ − 4ac)/2c. In that equation, a = − 0.41 × EM; b = 0.877 × EM + (0.41 × DMI) + EG; and c = − 0.877 × DMI, where EM = energy for maintenance and EG = energy for gain. Energy for maintenance (**EM**; Mcal/d) was estimated using EM = 0.077 × ABW^0.75^, where ABW = average BW × 0.96 (Lofgreen and Garrett, 1968) and energy gain (**EG**; Mcal/d) was estimated using the equation 0.0557 × EQSBW^0.75^ × ADG^1.097^, where EQSBW = ABW × 478/AFBW (Zinn and Shen, 1998; NASEM, 2016). Performance-calculated net energy of gain (**NEg**) was subsequently calculated from NEm (NEg = 0.877 × NEm − 0.41) as previously described by Zinn and Shen (1998). Both performance-calculated NEm and NEg were determined over the entire feeding period and included the transition period for the REC treatment.

Rumination and activity data were collected using three-axis accelerometer ear tags that provided data as the min spent active or ruminating within a 2 h interval. Time spent ruminating or active was averaged daily and multiplied by 12 to calculate the total min ruminating and active per day. Rumen boluses produced a single pH value every 10 min. Daily ruminal pH was calculated by averaging the pH values generated within a day. A pH of 5.6 was considered the threshold for ruminal acidosis (Cooper et al., 1998). It was assumed that if the pH reading was below the threshold of 5.6, the subsequent 10 min were also below 5.6. Daily time below a pH of 5.6 was calculated by summing the min below a pH of 5.6 within a day. To calculate the area under the curve (**AUC**) of 5.6, the pH value was subtracted from 5.6 and multiplied by 10 min each time it was below 5.6. The AUC was then summed within a day. Rumination and rumen pH data were only recorded through day 146 because of limitations in the battery life of the technology.

### Statistical analysis

Continuous data (performance and non-categorical carcass data) were analyzed as a generalized complete block design using the MIXED procedure of SAS (SAS Inst. Inc., Cary, NC). Dietary treatment was analyzed in the model as a fixed effect with block as the random effect. Ruminal pH, rumination behavior, total activity, and serum chemistry were analyzed as repeated measures using the MIXED procedure with the main effects of treatment, day, and their interaction determined for each repeated variable. For data analyzed using repeated measures, the subject was pen within the block. Covariance structure was selected by using the lowest Akaike information criterion value. Categorical data such as morbidity, mortality, quality grade, and liver score were analyzed using the GLMMIX procedure of SAS as binomial proportions. Treatment was included as a fixed effect in the model, whereas pen within treatment and block combination was used as a random effect. Treatment means are presented as least square means ± standard error of the mean (**SEM**). Statistical significance was declared at *P* ≤ 0.05, and a tendency was declared when 0.05 < *P* ≤ 0.10.

## Results and Discussion

### Live animal performance

From days 0 to 14, 14 to 28, 28 to 56, 56 to 74, and 0 to 74, DMI was less (*P* < 0.01; Table 2) for cattle consuming FIN compared with REC. The early differences in DMI translated to 0.26 kg/d less (*P* = 0.01) DMI for FIN from days 0 to final, although DMI after day 74 did not differ (*P* = 0.80) when both treatments were consuming the same finishing diet. Because the REC and FIN diets had different energy densities in the first 56 d, ME intake was also evaluated to compare differences in dietary energy consumption. ME intake was less for FIN cattle from days 0 to 14 (*P* < 0.01) and days 14 to 28 (*P* = 0.02), but not different (*P* ≥ 0.19) for any of the time periods evaluated after day 28.

**Table 2.** Dry matter intake of cattle fed a receiving or finishing diet upon arrival to the feedlot

Item	Treatments		SEM^3^	*P*-value
	REC^1^	FIN^2^		
Dry matter intake, kg/d				
Days 0 to 14	3.90	2.78	0.13	<0.01
Days 14 to 28	6.43	5.19	0.16	<0.01
Days 28 to 56	8.18	7.14	0.14	<0.01
Days 56 to 74	8.68	8.07	0.17	<0.01
Days 0 to 74	7.16	6.17	0.11	<0.01
Days 74 to final	8.99	9.02	0.11	0.80
Days 0 to final	8.48	8.22	0.10	0.01
Calculated ME intake^4^, Mcal/d				
Days 0 to 14	10.79	8.92	0.36	<0.01
Days 14 to 28	17.77	16.62	0.48	0.02
Days 28 to 56	22.60	22.86	0.40	0.51
Days 56 to 74	25.96	25.85	0.52	0.84
Days 0 to 74	20.19	19.77	0.31	0.19
Days 74 to final	28.79	28.88	0.36	0.80
Days 0 to final	26.41	26.32	0.30	0.78

REC, cattle fed a receiving diet for the first 56 d then transitioned to a finishing diet over 18 d.

FIN, cattle fed a finishing diet for the entire feeding period.

SEM, standard error of the mean.

Calcualated ME intake = metabolizable energy (ME) intake calculated by multiplying daily dry matter intake (DMI) by dietary ME.

A series of similar experiments were conducted at the Clayton Livestock Research Center (Clayton, NM) from 1975 to 1981 to evaluate the implications of dietary energy density and/or concentrate inclusion on growth performance and health of newly received feedlot calves. Lofgreen et al. (1975) evaluated diets containing 20.0%, 55.0%, 72.0%, or 90.0% concentrate in three experiments. Overall, DMI tended to decrease as the percentage concentration increased in the diet. In a subsequent study, Lofgreen et al. (1980) also observed less DMI in cattle consuming a 75.0% concentrate diet compared with a 50.0% and 25.0% concentrate diet. Similarly, close-out data from Iowa feedlots between 1988 and 1997 indicated cattle consuming a diet with >75.0% concentrate consumed 9.61 kg/d and cattle consuming <75.0% concentrate diets had 0.31 kg greater DMI (9.92 kg/d) over the entire feedlot period (Koknaroglu et al., 2005).

Greater DMI of REC in the present study may be from cattle eating to meet an energy requirement and subsequently consuming more of a less energy-dense diet. When ruminants consume forage-based diets, satiety signaling is likely dictated by gut fill as opposed to the chemical feedback that occurs with higher concentrate feedlot cattle diets with greater dietary energy density (Forbes, 2003). Therefore, when DMI is not limited by gut fill, cattle will consume more of a low energy diet to meet energy requirements for maintenance and growth and less of a high energy diet if they are eating to a constant energy intake. It was previously observed that when the energy in a feedlot diet is diluted by roughage concentration, cattle will increase DMI to match the energy intake of the undiluted diet (Price et al., 1980; Krehbiel et al., 2006). Alternatively, providing greater proportions of familiar feed ingredients may have increased DMI. Cattle arriving at the feedlot have likely been grazing or fed forage sources such as hay and would be more familiar with these ingredients compared with grain.

It is also important to note that the diets (Table 1) were not balanced for nutrient content to match industry standards for receiving and finishing diets. Therefore, in addition to energy concentration, the diets also differed in monensin, CP, and CF concentrations, which could also have affected DMI. For example, the FIN diet contained greater concentrations of monensin compared with REC (38.0 vs. 26.5 mg/kg). Monensin has been observed to reduce DMI (Duffield et al., 2012) and therefore, the higher concentration of monensin in FIN could have influenced DMI in this study. The FIN diet also contained less CP compared with the REC diet (13.2% vs. 13.9%). However, neither diet was considered deficient in CP according to NASEM (2016). In addition to differences in CP, corn oil was added to the FIN diet but not to the REC diet. Fat can limit DMI, but the inclusion level used in this study (3.0% of added corn oil on a DM basis) should not have negatively impacted DMI (Zinn, 1989) and was within the range of added fat recommended by feedlot consulting nutritionists for finishing cattle diets (Samuelson et al., 2016). Further research may be necessary to confirm that CP and CF concentrations did not contribute to the differences in DMI and performance during the receiving period.

BW of cattle consuming REC was 8 kg greater than FIN (Table 3; *P* < 0.01) on day 14. On day 28, BW of FIN and REC did not differ (266 kg; *P* = 0.87), and by day 56, BW of cattle fed FIN was 8 kg greater than REC (*P* = 0.01). On day 74, when REC cattle were completely transitioned to the finishing diet, the BW of FIN was 10 kg greater than REC (*P* < 0.01) and the final BW of FIN tended to be greater than REC (*P* = 0.10; 638 vs. 629 kg for FIN and REC, respectively). In the first 14 d, REC cattle gained 0.60 kg/d while FIN cattle had 0.0 kg ADG (*P* < 0.01). Although REC cattle had greater DMI during this period, when predicting ADG from DMI and dietary energy concentrations (NASEM, 2016; data not shown), REC cattle were expected to gain 0.28 kg/d while FIN cattle were predicted to gain 0.43 kg/d. Because REC cattle outperformed expected ADG and FIN cattle underperformed expected ADG, this suggests the differences in ADG and BW from days 0 to 14 could be influenced by differences in gut fill during realimentation after the marketing process. From days 14 to 28, FIN had greater (*P* < 0.01) ADG than REC. In addition, from days 28 to 56, FIN had greater (*P* < 0.01) ADG compared with REC with no difference (*P* = 0.24) in ADG from days 56 to 74. No difference in ADG among treatments from days 56 to 74 likely occurred because dietary composition became more similar over that time period as cattle fed REC was eating a portion of their diet as FIN. Over the first 74 d, FIN had 9.8% greater (*P* < 0.01) ADG than REC with no difference (*P* = 0.99) in ADG from d 74 to final, when all animals were consuming the same diet.

**Table 3.** Performance of cattle fed a receiving or finishing diet upon arrival to the feedlot

Item	Treatments		SEM^3^	*P*-value
	REC^1^	FIN^2^		
BW^4^, kg				
Day 0	242	242	0.3	0.26
Day 14	250	242	1.9	<0.01
Day 28	266	266	2.1	0.87
Day 56	313	321	2.6	0.01
Day 74	340	350	2.6	<0.01
Final	629	638	5.7	0.10
ADG^5^, kg				
Days 0 to 14	0.60	0.00	0.13	<0.01
Days 14 to 28	1.15	1.74	0.09	<0.01
Days 28 to 56	1.67	1.96	0.08	<0.01
Days 56 to 74	1.51	1.61	0.08	0.24
Days 0 to 74	1.33	1.46	0.05	<0.01
Days 74 to final	1.50	1.50	0.03	0.99
Days 0 to final	1.41	1.45	0.03	0.18
G:F^6^				
Days 14 to 28	0.181	0.338	0.015	<0.01
Days 28 to 56	0.204	0.274	0.010	<0.01
Days 56 to 74	0.175	0.199	0.009	0.01
Days 0 to 74	0.186	0.236	0.004	<0.01
Days 74 to final	0.167	0.166	0.002	0.80
Days 0 to final	0.166	0.176	0.003	<0.01

REC, cattle fed a receiving diet for the first 56 d then transitioned to a finishing diet over 18 d.

FIN, cattle fed a finishing diet for the entire feeding period.

SEM, standard error of the mean.

Shrunk body weight (BW) is reported as un-shrunk BW × 0.96.

ADG, average daily gain.

G:F, gain:feed.

Although there was less ME intake in the first 28 d and similar ME intake after day 28, the efficiency of ME utilization may have been different between dietary treatments and influenced ADG. Jennings et al. (2020) observed finishing cattle fed a 15.0% corn stalk diet consumed similar ME to cattle fed a 5.0% or 10.0% corn stalk diet, but had reduced ADG. Greater heat production from the fermentation of roughage may have reduced the efficiency of ME utilization by the animals consuming the REC diet (Lofgreen and Garrett, 1968; Reynolds et al., 1991) and allowed cattle fed the FIN diet to have greater ADG after day 14. Another explanation for the differences in performance despite no difference in ME intake is that the ME values reported for concentrate ingredients such as grains in the NASEM (2016) publication are underestimated. Updated equations have been proposed by Galyean et al. (2016) and Hales (2019) to account for differences in the conversion of digestible energy to ME between roughage and concentrate ingredients.

Because FIN did not gain BW during the first 14 d, G:F was not calculated for this period. However, from days 14 to 28, 28 to 56, 56 to 74, and 0 to 74, G:F was greater (*P* ≤ 0.01) for FIN because they consumed less feed and gained similarly to REC. Furthermore, increased G:F is from a combination of increased ME concentration and decreased DMI in FIN compared with REC cattle that allowed for similar ME intake and suggests greater ME utilization. After day 74, when all cattle were consuming the finishing diet and had similar DMI, there was no difference (*P* = 0.80) in G:F. However, the overall (days 0 to final) G:F for FIN was greater (*P* < 0.01) than REC, indicating that the early differences in G:F impacted overall feed efficiency.

### Health

Health outcomes are presented in Table 4. The proportion of cattle treated once or twice for BRD was 48.5% and 20.0%, respectively, for both treatments (*P* = 1.00). The percentage of cattle treated thrice for BRD was 10.0% and 10.5% for REC and FIN, respectively (*P* = 0.87). The proportion of cattle treated for illness not associated with BRD such as lameness, abscesses, and bloat did not differ (*P* = 0.31) between treatments. The mortality rate for REC and FIN was 1.5% and 3.0%, respectively, and was not different (*P* = 0.31). Therefore, the results of the present study suggest that feeding a high-energy finishing diet in lieu of a traditional receiving diet containing higher concentrations of roughage increases growth performance and feed efficiency, but does not alter the health of high-risk cattle. Previously, Lofgreen et al. (1975) concluded that feeding a 90.0% concentrate diet compared with a 72.0% or 55.0% concentrate diet increased morbidity of auction-derived steers. Rivera et al. (2005) later conducted a meta-analysis of data from the Clayton Livestock Research Center (Clayton, NM) and similarly reported a correlation between increased concentrate level and morbidity of feedlot cattle.

**Table 4.** Morbidity and mortality measurements for cattle fed a receiving or finishing diet upon arrival to the feedlot

Item	Treatments		SEM^3^	*P*-value
	REC^1^	FIN^2^		
1st BRD treatment^4^, %	48.5	48.5	-	1.00
2nd BRD treatment, %	20.0	20.0	-	1.00
3rd BRD treatment, %	10.0	10.5	-	0.87
Other treatment^5^, %	5.0	3.0	-	0.31
Mortality, %	1.5	3.0	-	0.31
Days to 1st treatment	16.2	17.8	2.95	0.58
Days to 2nd treatment	25.8	22.9	5.01	0.57
Days to 3rd treatment	32.5	31.7	5.67	0.88
Days to mortality	58.8	49.5	9.02	0.38

REC, cattle fed a receiving diet for the first 56 d then transitioned to a finishing diet over 18 d.

FIN, cattle fed a finishing diet for the entire feeding period.

SEM, standard error of the mean.

BRD, bovine respiratory disease.

Other treatments included bloat, lameness, or injury.

The discrepancy between the current research and that of Lofgreen et al. (1975) and Rivera et al. (2005) could be influenced by differences in diet formulations between studies. For example, steam-rolled barley was used by Lofgreen et al. (1975) and had greater ruminal starch digestibility compared with steam-flaked corn (Zinn, 1993). The diets used in the current study contained GMP, which may have reduced the rate of starch fermentation and mitigated the incidence of acidosis compared with Lofgreen et al. (1975). Because BRD and acidosis have similar clinical signs (i.e., depression, reduced or erratic DMI, poor body condition, etc.), decreasing the potential for acidosis may have reduced misdiagnosis of BRD and minimized differences in morbidity in the present study. In addition, the study conducted by Lofgreen et al. (1975) only used 107 steers with 2 pens per treatment and likely lacked statistical power to confidently delineate morbidity effects.

The number of days before cattle were treated once, twice, or thrice for BRD were not different (*P* ≥ 0.57) and the average number of days before mortality were not different (*P* = 0.38) between REC and FIN. Snowder et al. (2006) observed that the peak time of BRD morbidity was at 14 DOF, which is similar to the average days to first treatment (17 d) in the current study. Mortality rates in small pen research should be interpreted with caution because the occurrence is typically low and a single mortality event has the potential to greatly influence pen means within a small sample size.

### Growth characteristics

Ultrasound measurements were collected throughout the study to understand compositional changes in 12th rib fat thickness and rump fat thickness, ribeye area, and intramuscular fat over the feeding period. As expected, there was no difference (Table 5; *P* ≥ 0.65) in ultrasound measurements collected on day 0. By day 74, FIN had greater fat thickness over the 12th rib (*P* = 0.04) and rump (*P* = 0.04) and tended to have greater ribeye area (*P* = 0.10) with no difference in intramuscular fat (*P* = 0.93). However, by day 146, there was no difference (*P* ≥ 0.37) for any of the ultrasound measurements recorded. These results indicate that providing a diet with greater energy density earlier in the feeding period only had transient effects on body composition and does not cause excessive fat deposition in relation to lean tissue and bone growth.

**Table 5.** Ultrasound measurements of cattle fed a receiving or finishing diet upon arrival to the feedlot

Item	Treatments		SEM^3^	*P*-value
	REC^1^	FIN^2^		
12th rib fat thickness, cm				
Day 0	0.23	0.23	0.01	0.99
Day 74	0.45	0.49	0.02	0.04
Day 146	0.86	0.88	0.03	0.68
Rump fat thickness, cm				
Day 0	0.24	0.23	0.01	0.87
Day 74	0.59	0.64	0.02	0.04
Day 146	1.10	1.12	0.03	0.52
Ribeye area, cm^2^				
Day 0	44.45	44.15	0.66	0.65
Day 74	61.20	63.07	1.11	0.10
Day 146	81.76	82.67	1.01	0.37
Intramuscular fat, %				
Day 0	2.93	2.93	0.04	0.98
Day 74	3.14	3.14	0.04	0.93
Day 146	3.61	3.63	0.07	0.84

REC, cattle fed a receiving diet for the first 56 d then transitioned to a finishing diet over 18 d.

FIN, cattle fed a finishing diet for the entire feeding period.

SEM, standard error of the mean.

Klinger et al. (2007) observed similar results when limit-feeding a high energy diet (1.22 Mcal/kg NEg) compared with a high roughage diet (0.94 Mcal/kg NEg) during a 77-d backgrounding trial. Cattle fed the high energy diet had greater 12th rib fat on day 77 compared with ad libitum feeding of the low energy diet (Klinger et al., 2007). Wright and Russel (1991) enrolled cattle at 250 kg BW and provided either grass pellets at a high level of energy (0.05 Mcal/kg BW) throughout the trial, or a low level of energy (0.04 Mcal/kg BW) until they reached 350 kg, when both treatments were offered the same diet. During treatment application, cattle with greater energy intake also had greater body fat. However, similar to the current study, once dietary energy concentrations did not differ, the body composition of both groups was similar by the time each group reached 450 kg of live weight (Wright and Russel, 1991).

### Carcass characteristics

HCW (Table 6) of FIN cattle was 9 kg greater (*P* = 0.04) than REC (414 vs. 405 kg, respectively). However, the dressing percentage was not different (*P* = 0.11) between FIN (64.8%) and REC (64.5%) cattle, which suggests that the additional BW contributed by FIN was deposited primarily in the carcass. There was no difference in carcass 12th rib fat thickness (*P* = 0.46) or carcass ribeye area (*P* = 0.11) among cattle consuming FIN or REC. Furthermore, there was no difference in marbling score (*P* = 0.17) or quality grade (*P* ≥ 0.31). Calculated yield grade was also not different between treatments (*P* = 0.77). With the exception of greater HCW for FIN, similar carcass characteristics support the ultrasound observations on day 146 and illustrate that feeding a high-energy diet at arrival does not negatively impact carcass composition. Furthermore, the greater fat deposition measured via ultrasound at the beginning of the feeding period did not result in over fattening cattle before slaughter. Limited data exist evaluating the effects of dietary energy density during the receiving period on carcass characteristics of finishing cattle. Much of the previous research focusing on this topic (Lofgreen et al., 1975, 1980; Tomczak et al., 2019) has only been evaluated during the receiving period, from 28 to 56 d after arrival, and did not report differences in cattle performance and body composition over the entire feeding period or post-mortem carcass characteristics. However, when cattle were followed to slaughter by Lofgreen et al. (1975), there were no differences in carcass composition despite performance differences observed early in the feeding period.

**Table 6.** Carcass traits of cattle fed a receiving or finishing diet upon arrival to the feedlot

Item	Treatments		SEM^3^	*P*-value
	REC^1^	FIN^2^		
HCW^4^, kg	405	414	3.78	0.04
Dressing, %	64.5	64.8	0.20	0.11
12th rib fat thickness, cm	1.47	1.52	0.06	0.46
Ribeye area, cm^2^	94.7	96.4	1.03	0.11
Marbling score^5^	44.68	45.95	0.91	0.17
Quality grade, %				
Prime	1.54	1.56	-	0.98
Choice	66.68	70.54	-	0.43
Select	31.25	26.33	-	0.31
Standard	0.53	1.57	-	0.61
Calculated yield grade^6^	3.08	3.10	0.09	0.77
Liver abscesses, %	20.77	14.72	-	0.18

REC, cattle fed a receiving diet for the first 56 d then transitioned to a finishing diet over 18 d.

FIN, cattle fed a finishing diet for the entire feeding period.

SEM, standard error of the mean.

HCW, hot carcass weight.

Leading digit indicates marbling score: 2 = trace, 3 = slight, 4 = small, 5 = modest, 6 = moderate, 7 = slightly abundant, 8 = moderately abundant, 9 = abundant.

Calculated using the USDA (1997) regression equation.

Cattle fed the FIN diet had 14.7% liver abscesses and were not different (*P* = 0.18) from the REC treatment (20.8%). Liver abscesses are a concern in feedlot cattle because of the reduced performance and carcass value associated with their formation (Nagaraja and Chengappa, 1998). In previous research reported by Klinger et al. (2007), two trials were conducted to evaluate limit feeding on a high-energy diet vs. ad libitum feeding on a high roughage diet. There was no difference in the proportion of liver abscesses among treatments in the first trial, but a greater proportion of liver abscesses were observed in the limit-fed cattle in the second trial. When cattle are provided ad libitum feed following DMI restriction, they may overconsume and subsequently induce ruminal acidosis that could lead to liver abscesses. Therefore, Klinger et al. (2007) proposed the difference in liver abscess proportion between trials may have been from the more restricted DMI in trial 2 compared with trial 1. In the current study, the feed offered was managed similarly for both FIN and REC and could explain the lack of difference in liver abscesses observed in contrast to Klinger et al. (2007).

### Carcass-adjusted performance, body composition, and performance-calculated energy

Similar to final BW, there was no difference in carcass-AFBW (*P* = 0.46; Table 7) between REC (631 kg) and FIN (636 kg). In addition, carcass-adjusted ADG was not different (*P* = 0.47) between REC (1.46 kg) and FIN (1.47 kg). The lack of difference in carcass-adjusted ADG is not surprising because there was no difference in live ADG from days 0 to final. There was a tendency (*P* = 0.06) for carcass-adjusted G:F to be greater for FIN compared with REC and is likely from numerically greater carcass-adjusted ADG and less DMI from days 0 to final.

**Table 7.** Carcass-adjusted performance, empty body fat, empty body weight, adjusted final body weight, and performance calculated energy of cattle fed a receiving or finishing diet upon arrival to the feedlot

Item	Treatments		SEM^3^	*P*-value
	REC^1^	FIN^2^		
Carcass-adjusted performance				
Final body weight^4^, kg	631	636	6.15	0.46
ADG^5^, kg	1.46	1.47	0.02	0.47
G:F^6^	0.173	0.178	0.002	0.06
Empty body fat^7^, %	30.54	31.06	0.35	0.15
Empty body weight^8^, %	569	573	5.23	0.46
AFBW^9^, %	598	594	11.75	0.42
Performance-calculated NE^10^				
NEm, Mcal/kg	2.07	2.12	0.03	0.04
NEg, Mcal/kg	1.41	1.45	0.02	0.04

REC, cattle fed a receiving diet for the first 56 d then transitioned to a finishing diet over 18 d.

FIN, cattle fed a finishing diet for the entire feeding period.

SEM, standard error of the mean.

Carcass-adjusted final BW = HCW/0.6465 where BW = body weight and HCW = hot carcass weight.

Carcass-adjusted ADG = (carcass adjusted final BW − initial shrunk BW)/days on feed.

Carcass-adjusted G:F = carcass adjusted ADG/DMI where DMI = dry matter intake.

EBF = 17.76207 + (4.68142 × fat thickness) + (0.01945 × HCW) + (0.81855 × Quality grade) − (0.06754 × ribeye area) from Guiroy et al. (2001).

EBW = (1.316 × HCW) + 32.29 from Guiroy et al. (2001).

AFBW = adjusted final BW, AFBW = [EBW + (28 − EBF) × 14.26]/0.891 (Guiroy et al., 2001).

Performance-calculated dietary NEm = −b ± √(b^2^ − 4ac)/2c and performance-calculated dietary NEg = 0.877 × NEm − 0.41, where a=− 0.41 × EM; b = 0.877 × EM + (0.41 × DMI) + EG; and c = − 0.877 × DMI (Zinn and Shen, 1998). Energy maintenance (EM; Mcal/d) was estimated using EM = 0.077 × ABW^0.75^, where ABW = average BW × 0.96 (Lofgreen and Garrett, 1968) and energy gain (EG; Mcal/d) was estimated using the equation 0.0557 × (ABW × 478/AFBW^0.75^) × ADG^1.097^.

There was no difference in EBF between treatments (*P* = 0.15). Similarly, when EBW was calculated from HCW, there was no difference (*P* = 0.46) between treatments. Because there was a numerical trend for EBF to be greater for FIN, when calculated to a similar EBF, AFBW was not different (*P* = 0.42) between REC and FIN. Performance-calculated NE was greater (*P* = 0.04) for FIN than REC. This was expected because of the improvement in G:F for the FIN cattle compared with REC and further supports the hypothesis that cattle consuming FIN used ME more efficiently compared with the REC diet.

### Blood chemistry

There were no differences observed by dietary treatment for serum creatinine, K^+^, or total CO_2_ (*P* ≥ 0.27; Table 8). However, there was a diet × day interaction (*P* = 0.02) for BUN where FIN had reduced BUN compared with REC on day 28. BUN concentration decreased (*P* < 0.01; day effect) from day 0 (10.75 and 10.54 mg/dL for FIN and REC, respectively) to day 14 (7.67 and 8.67 mg/dL for FIN and REC respectively). Elevated BUN concentration of newly received cattle on day 0 may be caused by mobilization of protein stores to compensate for inadequate DMI (Richeson et al., 2015), or from immune requirements for amino acids (Petersen et al., 2004). Lower BUN for FIN compared with REC on day 28 is likely from the combination of less DMI, lower dietary CP, and potential differences in microbial utilization of rumen degradable protein because of greater ruminal availability of energy from the increased proportion of steam-flaked corn in FIN vs. REC (62.0% and 24.2% steam-flaked corn for FIN and REC, respectively). Ellenberger et al. (1989) observed that cattle had decreased BUN after refeeding from 50.0% of ad libitum DMI despite high protein intake, indicating the efficiency of protein use increases with energy consumption.

**Table 8.** Effects of diet on serum chemistry in newly received feedlot cattle

	Treatments			*P*-value		
Item	REC^1^	FIN^2^	SEM^3^	Diet	Day	Diet × day
Alanine transaminase, U/L						
Day 0	22.08	20.17	1.39	0.18	<0.01	0.49
Day 14	16.92	14.50				
Day 28	18.63	17.92				
Blood urea nitrogen, mg/dL						
Day 0	10.54	10.75	0.54	0.11	<0.01	0.02
Day 14	8.67	7.67				
Day 28^*^	8.21	6.33				
Cl^−^, mmol/L						
Day 0	99.63	98.54	0.69	0.37	0.03	0.03
Day 14^*^	96.88	98.50				
Day 28	97.38	98.54				
Creatinine, mg/dL						
Day 0	1.73	1.58	0.07	0.33	<0.01	0.22
Day 14	1.20	1.27				
Day 28	1.15	1.09				
Glucose, mg/dL						
Day 0	82.33	85.04	3.38	0.64	<0.01	0.73
Day 14	90.17	92.67				
Day 28	98.96	98.25				
K^+^, mmol/L						
Day 0	5.38	5.36	0.13	0.48	0.02	0.65
Day 14	5.70	5.52				
Day 28	5.66	5.58				
Na^+^, mmol/L						
Day 0	142.71	141.12	0.72	0.53	<0.01	< 0.01
Day 14	140.75	139.79				
Day 28	140.71	142.00				
Total CO_2_, mmol/L						
Day 0	25.67	24.42	0.47	0.27	<0.01	0.64
Day 14	27.33	26.96				
Day 28	27.29	27.13				

REC, cattle fed a receiving diet for the first 56 d then transitioned to a finishing diet over 18 d.

FIN, cattle fed a finishing diet for the entire feeding period.

SEM, standard error of the mean.

Treatments differ within day *P* ≤ 0.05.

A diet × day interaction (*P* = 0.03) was also observed for blood concentrations of Cl^−^. On day 14, serum Cl^−^ for FIN was greater than REC but did not differ on day 0 or 28. Although there was a diet × day interaction (*P* < 0.01) for Na^+^, there were no treatment differences within day. Previously, Apple et al. (1993) observed no difference in blood Na^+^ or Cl^−^ concentrations after sheep were exposed to 18 h of restraint and isolation stress. Cole et al. (1988) also observed no difference in serum Na^+^ concentrations after 24 h of transport stress in newly received calves.

There was a day effect (*P* < 0.01) for ALT concentration, which decreased from days 0 to 14. Alanine transaminase is a marker of liver function, and an increased concentration can indicate liver damage (Pagana and Pagana, 2013). In high-risk cattle, Smock et al. (2020) also observed a decline in ALT in the first 14 d after feedlot arrival. If liver function is reduced when high-risk cattle arrive at the feedlot, it is not surprising that blood glucose concentrations were also less on day 0 compared with day 14 (*P* < 0.01). Because cattle have little ability to absorb glucose directly from the feed, gluconeogenesis is an important function of the liver to maintain blood glucose concentrations (Young, 1977). In addition, fasting during transport could have resulted in the lower blood glucose concentrations observed on day 0 compared with days 14 and 28 (Young, 1977). This suggests that as cattle began consuming feed, they were able to overcome the negative energy balance from stress and inadequate nutrition before feedlot arrival. There were no treatment differences observed for ALT or glucose concentrations (*P* ≥ 0.18).

### Rumen pH and animal behavior

**Figure 1.**
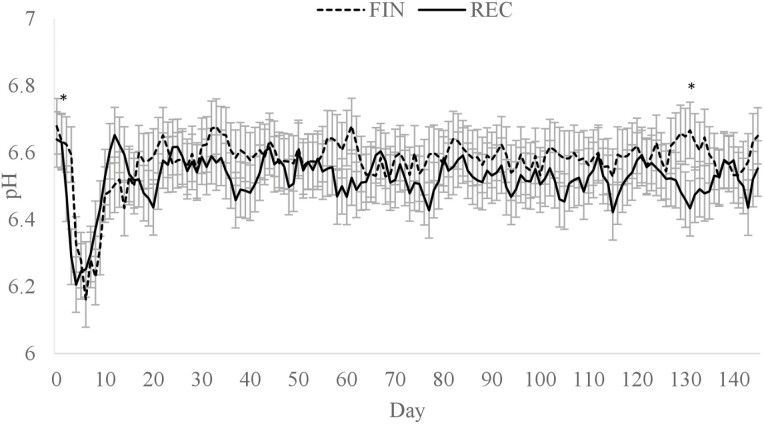
Mean daily ruminal pH of cattle fed a receiving or finishing diet upon arrival to the feedlot. Effect of diet, *P* = 0.62; day, *P* < 0.01; diet × day, *P* < 0.01. *Treatments differ within day, *P* ≤ 0.05.

**Figure 2.**
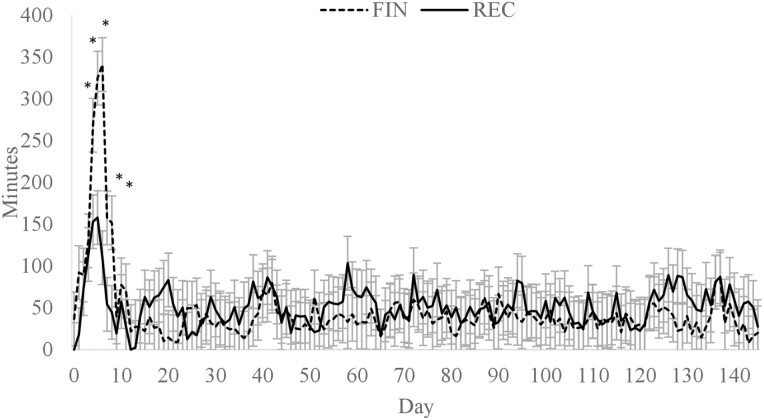
Minutes per day ruminal pH was below 5.6 for cattle fed a receiving or finishing diet upon arrival to the feedlot. Effect of diet, *P* = 0.86; day, *P* < 0.01; diet × day, *P* < 0.01. *Treatments differ within day, *P* ≤ 0.05.

**Figure 3.**
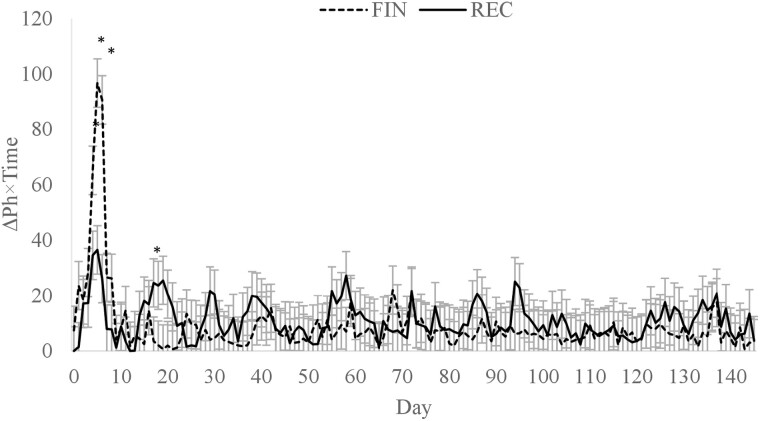
Daily area under a ruminal pH of 5.6 for cattle fed a receiving or finishing diet upon arrival to the feedlot. Effect of diet, *P* = 0.82; day, *P* < 0.01; diet × day, *P* = 0.03. *Treatments differ within day, *P* ≤ 0.05.

A diet × day interaction (*P* < 0.01; Figure 1) was observed for average daily ruminal pH where FIN had a greater rumen pH than REC on days 2 and 131, but no other time points differed. Greater rumen pH on day 2 was likely influenced by less DMI of the FIN cattle. However, by day 3, the ruminal pH of FIN cattle had declined to a similar level as REC. The difference observed on day 131 is likely from random variation and does not appear to have biological significance. Overall mean ruminal pH of REC and FIN were 6.52 and 6.57, respectively (*P* = 0.62). There was also a diet × day interaction (*P* < 0.01) for a time below a pH of 5.6 (Figure 2) and an area under a pH of 5.6 (Figure 3). Daily time below 5.6 was greater for FIN on days 4 to 8. Similarly, AUC for FIN was greater on days 4, 5, 6, and 19. A pH of 5.6 is considered the threshold for subacute ruminal acidosis in feedlot cattle (Owens et al., 1998). The peak time spent below pH of 5.6 was on day 6, where FIN spent approximately 341 min, and REC spent 154 min in a state of subacute acidosis. After day 8, the daily time below a pH of 5.6 did not exceed 85 min for either treatment.

In a similar study conducted with individually fed newly received calves, ruminal pH tended to be lower for cattle consuming a finishing diet than a receiving diet in the second week after arrival to the feedlot, but was not different at any other time (Tomczak et al., 2019). The greatest time below a pH of 5.6 and AUC did not occur until week 3 of the trial, where the time below reached 645 min per d for the finishing diet compared with 265 min for the receiving diet. Although a similar cattle source was used by Tomczak et al. (2019), the delayed reduction in ruminal pH compared with the current study may have been from differences in social dynamics that affect the eating behavior of cattle housed individually vs. in a group (González et al., 2008). In addition, Tomczak et al. (2019) reported that bunk management of the FIN diet was designed to allow a slower increase in DMI over the first 14 d that may have resulted in the restriction of DMI. After day 14, feed management changed, which allowed DMI to increase more rapidly compared with the first 14 d and may explain the increase in time below and AUC of 5.6 during week 3.

**Figure 4.**
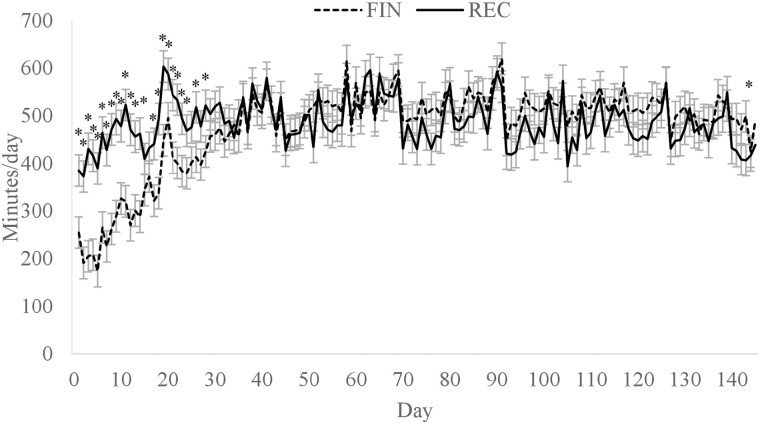
Minutes per day of rumination time for cattle fed a receiving or finishing diet upon arrival to the feedlot. Effect of diet, *P* = 0.67; day, *P* < 0.01; diet × day, *P* < 0.01. *Treatments differ within day, *P* ≤ 0.05.

**Figure 5.**
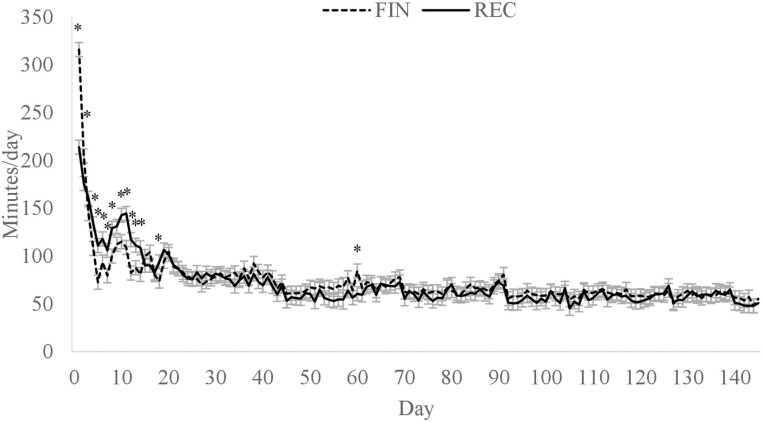
Minutes per day spent ruminating per kg of DMI for cattle fed a receiving or finishing diet upon arrival to the feedlot. Effect of diet, *P* = 0.61; day, *P* < 0.01; diet × day, *P* < 0.01. *Treatments differ within day, *P* ≤ 0.05.

**Figure 6.**
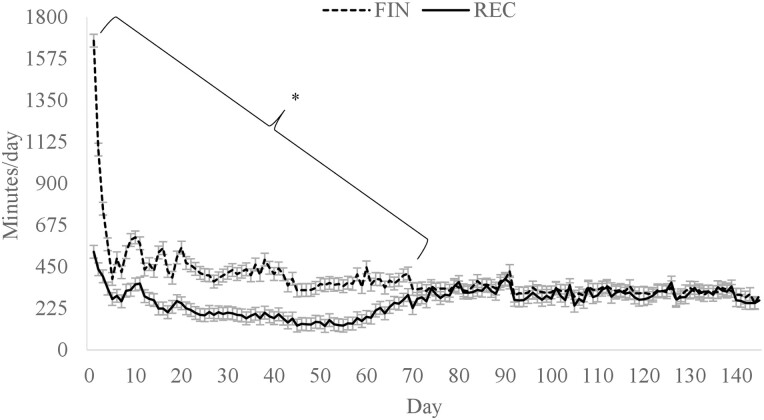
Minutes per day spent ruminating per kg of NDF for cattle fed a receiving or finishing diet upon arrival to the feedlot. Effect of diet, *P* < 0.01; day, *P* < 0.01; diet × day, *P* < 0.01. *Treatments differ within day, *P* ≤ 0.05.

There was a diet × day interaction for daily rumination min, where cattle assigned to REC spent more time ruminating on days 1 to 14, 17 to 23, 26, and 28 of the study (*P* < 0.01; Figure 4). Cattle in the REC group likely had greater rumination time because greater dietary roughage inclusion increases DMI and rumination (Galyean and Defoor, 2003). However, after day 28, there was no difference in rumination time except on day 143. Interestingly, the difference in rumination min was not present from days 28 to 56 despite the REC cattle consuming more feed and a greater proportion of roughage than the FIN cattle. When rumination time was expressed per kg of DMI, there was a diet × day interaction (*P* < 0.01; Figure 5) where the FIN cattle had greater rumination per kg DMI on days 1, 2, and 60 but less on days 4 to 8, 10 to 14, and 18. Tomczak et al. (2019) also observed that cattle consuming a receiving diet had greater rumination per kg DMI at the beginning of the feeding period compared with a finishing diet. The decreased time spent ruminating per kg of DMI in the present study on days 4 to 8, 10 to 14, and 18 could be from the lower inclusion of roughage in the FIN diet. However, when expressed as rumination per kg of NDF intake (Figure 6), rumination minutes was greater (*P* < 0.01) for cattle consuming FIN than REC from days 0 to 70. These results suggest that factors independent of diet may influence rumination time. Although there were differences in rumination, there was no dietary effect on animal activity (*P* = 0.48; data not shown).

### Implications

Providing a high-energy finishing diet upon arrival may be one option to increase growth performance and feed efficiency without impacting health. The results of this study also indicate that there may be a greater risk for ruminal acidosis in the first week after feedlot arrival, despite low DMI within both treatments. While the time below a pH of 5.6 was greater for FIN from days 4 to 8, the increased risk of acidosis in the first week after arrival did not affect the long-term growth performance of high-risk calves. High-risk cattle that enter the feedlot with unknown pre-arrival management have low DMI and increased risk for disease in the first 28 d. For this reason, there is potential to use alternative cattle feeding and management strategies to help facilitate improved performance in this type of cattle. Alternatively, for cattle with greater DMI, it may increase the risk for ruminal acidosis and negatively affect cattle performance. Additional research is also needed to investigate this concept in larger pens where differences in stocking density and feeding behavior may influence cattle performance, health, and the rumen environment.

## Abbreviations

ADF: acid detergent fiber
ADG: average daily gain
AFBW: adjusted final body weight
ALT: alanine aminotransferase
BRD: bovine respiratory disease
BUN: blood urea nitrogen
BW: body weight
CIS: clinical illness score
CP: crude protein
DM: dry matter
DMI: dry matter intake
DOF: days on feed
EBF: empty body fat
EBW: empty body weight
EE: ether extract
EG: energy for gain
EM: energy for maintenance
EQSBW: equivalent shrunk body weight
G:F: gain:feed
HCW: hot carcass weight
KPH: kidney-pelvic-and-heart fat
ME: metabolizable energy
NEg: net energy of gain
NEm: net energy of maintenance
OM: organic matter
